# 22q11.2 Deletion Syndrome: Influence of Parental Origin on Clinical Heterogeneity

**DOI:** 10.3390/genes15040518

**Published:** 2024-04-21

**Authors:** Melissa Bittencourt de Wallau, Ana Carolina Xavier, Carolina Araújo Moreno, Chong Ae Kim, Elaine Lustosa Mendes, Erlane Marques Ribeiro, Amanda Oliveira, Têmis Maria Félix, Agnes Cristina Fett-Conte, Luciana Cardoso Bonadia, Gabriela Roldão Correia-Costa, Isabella Lopes Monlleó, Vera Lúcia Gil-da-Silva-Lopes, Társis Paiva Vieira

**Affiliations:** 1 Medical Genetics and Genomic Medicine, Department of Translational Medicine, School of Medical Sciences, State University of Campinas, Campinas 13083-887, São Paulo, Brazil; m235500@dac.unicamp.br (M.B.d.W.); carolinaaraujomoreno@yahoo.com.br (C.A.M.); bonadia@unicamp.br (L.C.B.); groldao@unicamp.br (G.R.C.-C.); vgslopes@unicamp.br (V.L.G.-d.-S.-L.); 2 Centrinho Prefeito Luiz Gomes, Joinville 89203-020, Santa Catarina, Brazil; anacarolxavier@yahoo.com.br; 3 Instituto da Criança, Hospital de Clínicas, FMUSP, São Paulo 05403-000, São Paulo, Brazil; chong.kim@hc.fm.usp.br; 4 Serviço de Genética do Hospital de Clínicas da UFPR, Curitiba 80060-900, Paraná, Brazil; laine_med@yahoo.com.br; 5 Serviço de Genética do Hospital Infantil Albert Sabin—HIAS, Fortaleza 60410-794, Ceará, Brazil; erlaneribeiro@yahoo.com.br; 6 Centro de Atenção aos Defeitos da Face—CADEFI, Recife 50060-293, Pernambuco, Brazil; mandafono@hotmail.com; 7 Serviço de Genética Médica do Hospital de Clínicas de Porto Alegre—HCPA, Porto Alegre 90035-903, Rio Grande do Sul, Brazil; tfelix@hcpa.edu.br; 8 Serviço de Genética da Faculdade de Medicina de São José do Rio Preto (FAMERP/FUNFARME), São José do Rio Preto 15090-000, São Paulo, Brazil; genetica@famerp.br; 9 Serviço de Genética Médica do Hospital Universitário Prof. Alberto Antunes (HUPAA), Faculdade de Medicina, Universidade Federal de Alagoas (UFAL), Maceió 57072-900, Alagoas, Brazil; isabella.monlleo@gmail.com

**Keywords:** 22q11.2 deletion syndrome, clinical heterogeneity, parental origin, genomic imprinting

## Abstract

22q11.2 deletion syndrome (22q11.2DS) shows significant clinical heterogeneity. This study aimed to explore the association between clinical heterogeneity in 22q11.2DS and the parental origin of the deletion. The parental origin of the deletion was determined for 61 individuals with 22q11.2DS by genotyping DNA microsatellite markers and single-nucleotide polymorphisms (SNPs). Among the 61 individuals, 29 (47.5%) had a maternal origin of the deletion, and 32 (52.5%) a paternal origin. Comparison of the frequency of the main clinical features between individuals with deletions of maternal or paternal origin showed no statistically significant difference. However, *Truncus arteriosus*, pulmonary atresia, seizures, and scoliosis were only found in patients with deletions of maternal origin. Also, a slight difference in the frequency of other clinical features between groups of maternal or paternal origin was noted, including congenital heart disease, endocrinological alterations, and genitourinary abnormalities, all of them more common in patients with deletions of maternal origin. Although parental origin of the deletion does not seem to contribute to the phenotypic variability of most clinical signs observed in 22q11.2DS, these findings suggest that patients with deletions of maternal origin could have a more severe phenotype. Further studies with larger samples focusing on these specific features could corroborate these findings.

## 1. Introduction

The 22q11.2 region is one of the most complex regions of the human genome, because it contains several low copy repeats (LCRs). The presence of LCRs in this region increases its susceptibility to non-allelic homologous recombinations (NAHRs), leading to either the deletion or duplication of this genomic segment [1,2]. 22q11.2 deletion syndrome (22q11.2DS) is the most prevalent deletion syndrome, occurring at an estimated incidence of one in every 3000 to 6000 live births and one in every 1000 pregnancies [3,4].

Within the 22q11.2 region, there are 44 known protein coding genes, and the dosage imbalance of these genes may contribute to the development of the clinical manifestations of the syndrome. For instance, the *TBX1* gene, which plays a significant role in neural crest cell migration, is expressed in cells within the embryonic pharyngeal apparatus, which in turn transmit signals to neural crest cells. These cells are crucial for the development of several structures, including the craniofacial region, thymus, parathyroid glands, aortic arch, and cardiac outflow tract. These neural crest cells establish the vasculature of the pharyngeal arch arteries and the capsule of the thymus. Therefore, the deregulation of the *TBX1* gene affects neural crest migration within the pharyngeal region, impacting the morphogenesis of various structures, such as the sympathetic nervous system, skin, craniofacial skeleton, and aortic arch, all of which can be affected in 22q11.2DS [5,6,7,8].

The main clinical features of 22q11.2DS are: congenital heart disease, primarily conotruncal malformations such as ventricular septal defect, tetralogy of Fallot, interrupted aortic arch, and truncus arteriosus; palatal abnormalities, including velopharyngeal insufficiency, submucosal cleft palate, bifid uvula, and cleft palate; immunodeficiency, main due to thymic hypoplasia; facial dysmorphisms such as elongated face, low-set ears, high and broad nasal bridge, bulbous nasal tip, alar hypoplasia, hypertelorism, hooded eyelids, small mouth, and micro/retrognathia; and neurodevelopmental delay, especially speech delay and learning difficulties during childhood [9,10]. However, none of these phenotypes is observed in 100% of the patients, and the syndrome exhibits significant clinical heterogeneity, with more than 180 clinical manifestations already described, affecting various systems and organs [9,11].

The reason for this phenotypic variability is not well understood. The NAHRs events in the 22q11.2 region, between different LCRs, lead to deletions of variable sizes, containing different genes. 22q11.2DS includes the deletions that encompass the most proximal region, between LCRs A and D, including the *TBX1* gene. Most patients with 22q11.2DS harbor a deletion between LCRs A and D. However, other deletions between LCRs A and B or A and C, or other nested deletions can also be found. Several studies have explored the influence of deletion size on clinical variability, and most of them have failed to establish a significant association. This suggests that the main genes responsible for the clinical manifestations likely lie within the minimum region of overlap among the common deletions, between LCRs A and B, implying the presence of modifying factors elsewhere in the genome [1,12,13].

Other studies have focused on the influence of copy number variations (CNVs) and other variants, outside the 22q11.2 region, on the clinical heterogeneity of the syndrome. Smyk et al. (2023) [14] reported that 6.3% of individuals with 22q11.2DS had additional CNVs likely contributing to the clinical presentation. Zhao et al. (2023) [15] demonstrated that disruptions in chromatin regulatory genes affect the *TBX1* gene network, suggesting shared mechanisms between the *TBX1* gene network and the etiology of congenital heart diseases (CDH). Additionally, some studies have provided compelling evidence indicating that microRNA dysregulation is implicated in the development of schizophrenia in 22q11.2DS patients [16,17].

Beyond genetic factors, environmental effects, such as teratogen exposure during pregnancy, have also been considered as a potential phenotype modifier. However, most fetuses with 22q11.2DS do not experience such exposure, leaving the heterogeneity unexplained. Moreover, advancements in DNA and RNA sequencing approaches have unveiled epigenetic effects as modifiers in 22q11.2DS, exerting a significant impact on disease penetrance and severity [2,6,13]. In the 22q11.2 region, imprinting was described on the genes *DGCR6* and *DGCR6L* [18,19]. These genes, which down-regulate the *TBX1* gene, play a role in influencing neural crest migration within the pharyngeal region. Additionally, Chakraborty et al. (2012) [20] observed that dysregulation of *DGCR6* and *DGCR6L* was associated with psychopathological outcomes in children with 22q11.2DS [6,13].

A few previous studies have investigated the contribution of parental origin of the deletion to the clinical heterogeneity of 22q11.2DS. A study conducted by Seaver et al. (1994) suggested a potential impact of the maternal origin of the deletion on the presence of pulmonary atresia [21]. Another study, conducted by Eliez et al. (2001), indicated that the parental origin of the deletion could significantly affect brain development and morphology, with reduction in gray matter development attributed to presence of a 22q11.2 microdeletion on the maternal chromosome [22]. More recently, McGinn et al. (2022) investigated the influence of the parent of origin on intellectual outcomes in patients with 22q11.2DS and found no significant difference in full-scale IQ (FSIQ) based on the parental origin of *de novo* deletions [23].

Considering the limited number of studies analyzing the influence of the parental origin of the deletion on clinical heterogeneity, and that all of them compared parental origin with only one clinical feature, the aim of this study was to investigate the association between the clinical heterogeneity of 22q11.2DS and the parental origin of the deletion.

## 2. Materials and Methods

### 2.1. Patients

The patients were recruited from nine centers (medical genetics services or craniofacial rehabilitation centers) from different Brazilian regions, through a collaborative research of the Brazil’s Craniofacial Project (BCFP). The clinical data were previously collected through the Brazilian Database on Craniofacial Anomalies (BDCA) of the BCFP. A total of 61 unrelated patients with the 22q11.2DS, 35 female and 26 male, were included. The age at diagnosis varied from eight months to 32 years (mean age 10.4 years). Only patients with clinical data and DNA samples available of at least one parent were included.

All patients had a proximal 22q11.2 deletion, previously detected by multiplex ligation-dependent probe amplification (MLPA), with the P250 kit, or fluorescence in situ hybridization (FISH) with the TUPLE1 probe. Among the 57 individuals screened by MLPA, three had a deletion of approximately 1.5 Mb, between LCRs A and B, and 54 had a deletion of approximately 3 Mb, between LCRs A and D. Both types of deletions were included in the study as there is not a previous correlation established between deletion size and the clinical variability of 22q11.2DS. Individuals with central or distal deletions, not including the region between LCRs A and B, or inherited deletions were excluded.

This study received ethical approval by the Research Ethics Committee of the State University of Campinas (CAAE 47787521.6.0000.5404), and the inclusion of all participants occurred after the signing of the Informed Consent Form by one of their legal guardians.

### 2.2. Parental Origin Determination

The genomic DNA of the patients and their parents was obtained from peripheral blood samples, using a phenol–chloroform extraction method, with a standard protocol. The parental origin of the deletion was determined through genotyping of the microsatellite DNA markers D22S1638, D22S941, D22S944, and D22S1623, mapped within LCRs A and B, and D22S264, mapped within LCRs B and C. Genotyping of the single-nucleotide polymorphisms (SNPs) rs4819519 and rs5993650, mapped within LRCs A and B, was also performed. 

Genotyping of the microsatellite DNA markers was performed by polymerase chain reaction (PCR), with a standard protocol, and the amplified products were detected by capillary electrophoresis (3500xl Genetic Analyzer^®^, Applied Biosystems—Waltham, MA, USA). These results were analyzed by the online software Peak Scanner (3.1.1-PRC-build07, Thermo Fisher Connect™, Carlsbad, CA, USA) (Supplementary Figure S1). Genotyping of the SNPs rs4819519 and rs5993650 was performed by real-time PCR, using the Taqman assay and the ABI 7500 (Applied Biosystems—Waltham, MA, USA). The results were analyzed using the online software Standard Curve (2019.4.3-Q4-19-build6, Thermo Fisher Connect™, Carlsbad, CA, USA) (Supplementary Figure S2).

**Figure 1:**
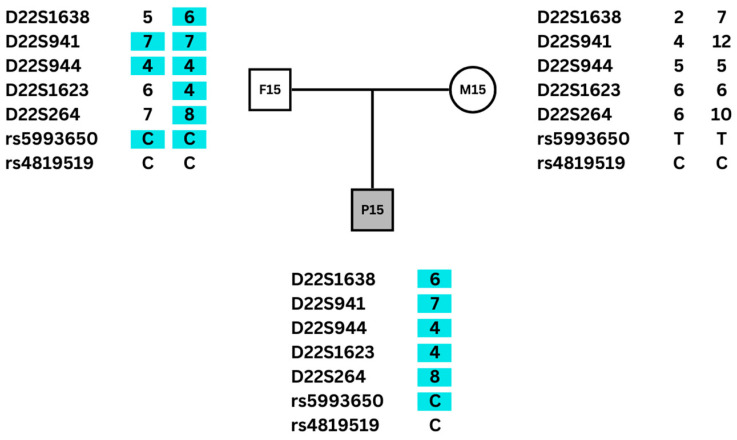
Alleles of DNA microsatellite markers and SNPs of a patient with 22q11DS and their parents. Only one allele was detected in the patient because the other was deleted. The proband shares five microsatellites and one SNP allele only with the father (highlighted in blue), so the deletion was determined to be of maternal origin. The rs4819519 was not informative in this family.

The alleles of microsatellite DNA markers and SNPs for each trio (proband, mother, and father) or pair (proband and mother) were established and compared on a spreadsheet (Supplementary Table S1). Parental origin was defined based on analysis of trios for 47 patients, and of pairs for 14 patients. The strategy of defining parental origin also with data from pairs (patient and mother) was chosen based on a previous study of the parental origin of 22q11.2DS [24]. Paternal origin was established in cases of trios in which the proband shared at least two alleles with the mother. Conversely, when the proband shared at least two alleles with the father, maternal origin was established. For the pairs, the paternal origin of the deletion was established when the proband shared all the analyzed alleles with the mother, while maternal origin was determined in cases where the proband did not share at least two alleles with the mother. Parental origin was defined based on three or more informative markers for 55 patients. In six cases, there were two informative markers (Supplementary Table S1). An example of the parental origin determination in a trio is shown in Figure 1.

### 2.3. Statistical Analysis

The patients were categorized into two groups based on the parental origin of the deletion (maternal or paternal), and the frequency of each clinical feature was compared between these groups. For statistical analysis, a Chi-square test was performed with a significance level of 5%, given that there were multiple clinical features to be compared as categorical variables. However, for clinical features with less than five individuals in one of the categories, the Fisher’s exact test was employed. In addition, a measure of effect size was estimated using Cramer’s V coefficient. These results were presented in the order 2 × 2, and the coefficient varied from −1 to +1, and were interpreted as follows: 0 < V ≤ 0.2 (weak association), 0.2 < V ≤ 0.6 (moderate association), and V > 0.6 (strong association). All the statistical tests were conducted using the SAS System for Windows (Statistical Analysis System—version 9.4—SAS Institute Inc., 2002–2008, Cary, NC, USA).

## 3. Results

**Figure 2:**
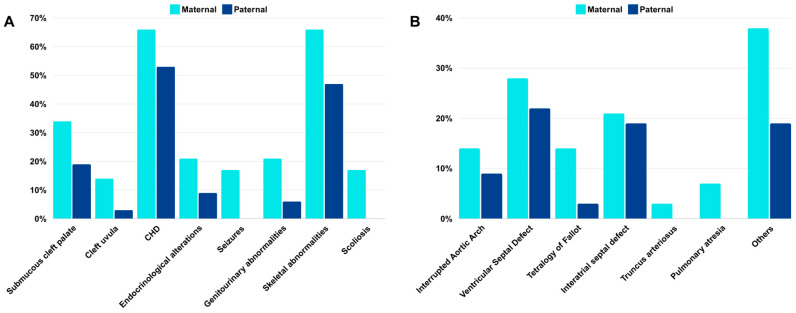
(**A**) Frequencies of selected clinical features with slight differences between patients with deletions of maternal (light blue) and paternal (dark blue) origin. (**B**) Frequencies of selected types of CHD between patients with deletions of maternal (light blue) and paternal (dark blue) origin.

Among the 61 patients, 29 (47.5%) had a 22q11.2 deletion of maternal origin and 32 (52.5%) showed a deletion of paternal origin (*p* = 0.7009). The main clinical features found in this sample were palatal abnormalities (77%; paternal 78%, maternal 76%), congenital heart diseases (CHDs) (59%; paternal 53%, maternal 66%), immunological or hematological abnormalities (57%; paternal 63%; maternal 52%), neurodevelopmental delay (79%; paternal 78%, maternal 79%), skeletal abnormalities (56%; paternal 47%, maternal 66%), and facial dysmorphisms (95%; paternal 91%, maternal 100%) (Table 1). No statistically significant difference was observed for the frequency of the main clinical manifestations between the groups with the 22q11.2 deletion of maternal or paternal origin, except for seizures (*p* = 0.0455) and scoliosis (*p* = 0.0200), which were found only in patients with deletions of maternal origin (Table 1, Figure 2A).

Although no statistically significant differences were found for most of the clinical features, there was a slight difference in the frequency of some of them between patients with deletions of paternal or maternal origin, including submucous cleft palate, cleft uvula, CHD, endocrinological alterations, genitourinary, and skeletal abnormalities, all of them more are frequently found in patients with the deletion in the maternal chromosome 22 (Table 1, Figure 2A). Among the CHD, tetralogy of Fallot (TOF) was more frequent in patients with deletions of maternal origin, and truncus arteriosus (TA) and pulmonary atresia were found only in patients with deletions in the maternal chromosome 22 (Figure 2B). Regarding endocrinological alterations, the most frequent were hypothyroidism, hypoparathyroidism, and hypocalcemia. Hypothyroidism was found in one patient with deletion of paternal origin and three patients with deletions of maternal origin, hypoparathyroidism in two patients (one paternal and one maternal), and hypocalcemia in another two patients (one paternal and one maternal). Among the genitourinary malformations, renal abnormalities were the most common findings, present in two patients with deletions of paternal origin, and in two patients with deletions of maternal origin.

## 4. Discussion

The phenotype of patients with 22q11.2DS is highly variable, and, to date, the reasons for that are not well understood [6,14]. Although a major contribution of parental origin on the phenotype is not expected in the 22q11.2 region, very few studies in the literature have compared the clinical features of patients with deletions of maternal or paternal origin [21,22,23]. Previous studies described random imprinting of the genes *DGCR6* and *DGCR6L*, which are mapped within the most common region deleted in the 22q11.2DS (between LCRs A and D) [18,19]. A further study conducted by Chakraborty et al. (2012) showed no evidence of parent-of-origin-related differences in the expression of either *DGCR6* or *DGCR6L*. However, they found a significantly greater variability in *DGCR6* expression in patients with 22q11DS than in age- and gender-matched control individuals. Therefore, they suggested that epigenetic mechanisms other than imprinting could contribute to the dysregulation of these genes in individuals with 22q11DS [20].

In the present study, the 22q11.2 deletion was of paternal origin in 52% of the patients and of maternal origin in 48%. To date, the largest study that investigated the parental origin of 22q11.2DS, conducted by Delio et al. (2013) [24], showed a statistically significant bias for maternal origin. They investigated 389 individuals with 22q11.2DS and found maternal origin of the deletion in 56% of them. In addition, they combined their results with previous studies and found that 57% of the patients had a 22q11.2 deletion of maternal origin, amounting to a ratio of 1.35 or a 35% increase in maternal compared to paternal origin [24]. Probably, the finding of 52% of the deletions being of paternal origin in the present study is by chance, due to a small sample size (61 individuals).

Regarding the influence of parental origin on clinical features, although there were no statistically significant differences for most of the clinical features between individuals with deletions of paternal or maternal origin, slight differences in the frequency of some features were found. These clinical signs included submucous cleft palate (19% paternal; 34% maternal), cleft uvula (3% paternal; 14% maternal), congenital heart diseases (53% paternal; 66% maternal), endocrinological alterations (9% paternal; 21% maternal), genitourinary alterations (6% paternal; 21% maternal), and skeletal abnormalities (47% paternal; 66% maternal) (Figure 2). Interestingly, all of them are slightly more frequent when the deletion is of maternal origin. In addition, *Truncus arteriosus*, pulmonary atresia, seizures (*p* = 0.0455), and scoliosis (*p* = 0.0200) were only found in patients with deletions of maternal origin. However, there were very few patients presenting with these features, and these results should be interpreted with caution. Although, these findings could suggest that patients with deletions of maternal origin would have a more severe phenotype. This could happen due to epigenetic mechanisms other than imprinting. However, one limitation of this study is the small sample size, and further studies with larger samples, including expression and methylation analyses, would be necessary to better clarify these findings.

Some previous studies have compared specific clinical signs with the parental origin of the deletion. A study by Seaver et al. (1994), which determined the parental origin of four patients with 22q11.2DS, showed that all of them had pulmonary atresia and a maternal origin of the deletion [21]. In the present study, pulmonary atresia was found in only two patients, both with a maternal deletion. However, both sample sizes are too small to draw any conclusion, and studies with larger samples are necessary. Another study, conducted by Eliez et al. (2001), investigated the influence of the parental origin of the deletion on the brain development of 18 individuals with 22q11.2DS. They found a 9% reduction in the total volume of gray matter in patients with maternal deletion. Nevertheless, in the current study, only two individuals had a brain MRI, both with normal results, and no comparisons between these studies are possible [22].

More recently, McGinn et al. (2022) conducted a study examining the association between parental origin and the full-scale intelligence quotient (FSIQ) in 81 individuals with de novo 22q11.2DS. Their research did not identify significant parent-of-origin differences in FSIQ for de novo deletions [23]. However, in the present study, data of FSIQ were not available for most of the patients. Although 78% of this cohort presented with developmental delay, only six patients had an established intellectual disability (three paternal; three maternal) and another 22 had suggestive findings of intellectual disability (11 paternal; 11 maternal) with no formal evaluation.

## 5. Conclusions

In this study, 52% of the patients had a 22q11.2 deletion of paternal origin and 48% of maternal origin. No statistically significant differences were found for most of the clinical features between patients with deletions of paternal or maternal origin. However, a slight difference in frequency was found for some clinical features, all of them more common in patients with deletions of maternal origin, and some features were found only in patients with a maternal-origin deletion. Although the parental origin of the deletion does not seem to have a major contribution to the phenotypic variability observed in 22q11.2DS, these findings suggest that patients with deletions of maternal origin could have a more severe phenotype. Further studies with larger samples will be necessary to better clarify the contribution of parental origin to the clinical heterogeneity of 22q11.2DS.

## Figures and Tables

**Table 1:** Clinical features found in patients with deletions of maternal or paternal origin.

	Paternal Origin		Maternal Origin		Total	*p*-Value	Cramer’s V
**Palatal abnormalities**	25/32	78%	22/29	76%	47/61 (77%)	0.4688	0.1107
Cleft palate	6/32	19%	6/29	21%	12/61 (20%)	0.9432	−0.0096
Submucous cleft palate	6/32	19%	10/29	34%	16/61 (26%)	0.1708	−0.1864
Velopharyngeal insufficiency	12/32	38%	11/29	38%	23/61 (38%)	0.9675	0.0056
Cleft uvula	1/32	3%	4/29	14%	5/61 (8%)	0.1842	−0.2036
**Congenital heart diseases (CHD)**	17/32	53%	19/29	66%	36/61 (59%)	0.3592	−0.1225
Interrupted aortic arch	3/32	9%	4/29	14%	7/61 (11%)	0.7040	−0.0631
Ventricular septal defect	7/32	22%	8/29	28%	15/61 (25%)	0.6956	−0.0537
Tetralogy of fallot	1/32	3%	4/29	14%	5/61 (8%)	0.1917	−0.1998
Atrial septal defect	6/32	19%	6/29	21%	12/61 (20%)	0.9408	−0.0102
*Truncus arteriosus*	0/32	-	1/29	3%	1/61 (1.6%)	-	-
Pulmonary atresia	0/32	-	2/29	7%	2/61 (3.2%)	-	-
Other CHD	6/32	19%	11/29	38%	17/61 (28%)	0.1173	−0.2151
**Immunological or hematological abnormalities**	20/32	63%	15/29	52%	35/61 (57%)	0.6497	0.0649
Recurrent infections	16/32	50%	14/29	48%	30/61 (49%)	0.8811	−0.0216
**Endocrinological alterations**	3/32	9%	6/29	21%	9/61 (15%)	0.6785	−0.1309
**Neurodevelopmental delay**	25/32	78%	23/29	79%	48/61 (79%)	0.7049	0.0615
Motor delay	12/32	38%	11/29	38%	23/61 (38%)	0.9890	0.0021
Language delay	20/32	63%	15/29	52%	35/61 (57%)	0.2724	0.1923
Behavioral delay	9/32	28%	8/29	28%	17/61 (28%)	0.9438	0.0106
**Behavioral/psychiatric and neurological alterations**	9/32	28%	9/29	31%	18/61 (30%)	0.9031	0.0181
ADHD	5/32	16%	4/29	14%	9/61 (15%)	1.0000	0.0258
Seizures	0/32	-	5/29	17%	5/61 (8%)	**0.0455**	−0.4016 *
**Hearing impairment**	9/32	28%	7/29	24%	16/61 (26%)	0.9805	0.0035
Sensorineural	1/32	3%	0/29	-	1/61 (1.6%)	-	-
Conductive	6/32	19%	4/29	14%	10/61 (16%)	0.7348	0.0669
**Ophthalmological abnormalities**	6/32	19%	7/29	24%	13/61 (21%)	0.4154	−0.1321
**Abnormalities of the genitourinary tract**	2/32	6%	6/29	21%	8/61 (13%)	0.2579	−0.2010
**Abnormalities of the gastrointestinal tract**	11/32	34%	11/29	38%	22/61 (36%)	0.8554	0.0292
**Skeletal abnormalities**	15/32	47%	19/29	66%	34/61 (56%)	0.2279	−0.1570
Long fingers	11/32	34%	10/29	34%	21/61 (34%)	0.8610	0.0228
Scoliosis	0/32	-	5/29	17%	5/61 (8%)	**0.0200**	−0.3139 *
**Facial dysmorphisms**	29/32	91%	29/29	100%	58/61 (95%)	-	-
Microcephaly	2/32	6%	2/29	7%	4/61 (7%)	1.0000	−0.0089
Long face	12/32	38%	15/29	52%	27/61 (44%)	0.3112	−0.1307
Hypertelorism	5/32	16%	3/29	10%	8/61 (13%)	0.7079	0.0850
Hooded eyelids	10/32	31%	14/29	48%	24/61 (39%)	0.2057	−0.1634
Typical nose	16/32	50%	16/29	55%	32/61 (52%)	0.7824	−0.0357

* Cramer’s V coefficient indicating moderate association.

## Data Availability

The data that support the findings of this study are available from the corresponding author upon reasonable request.

## Supplementary Materials

The following supporting information can be downloaded at: https://www.mdpi.com/article/10.3390/genes15040518/s1, Supplementary Table S1: Parental-origin definition by genotyping of DNA microsatellite markers and SNPs; Supplementary Figures S1 and S2: Graphic result examples for microsatellite DNA markers and SNPs of one of the families of this study.
