# Supplementary material for: 22q11.2 Deletion Syndrome: Influence of Parental Origin on Clinical Heterogeneity
Source: Genes (Basel). 2024 Apr 21;15(4):518. doi: 10.3390/genes15040518 (PMC11050591; doi:10.3390/genes15040518)
Supplement: Supplementary file 1 [file genes-15-00518-s001.zip › genes-2953481-supplementary.pdf]

SSupplemental material

22q11.2 deletion syndrome: evidence of no influence of parental origin on clinical heterogeneity

Graphic result examples for microsatellite DNA markers and SNPs of one of the families (patient, mother, and father) is showed below.

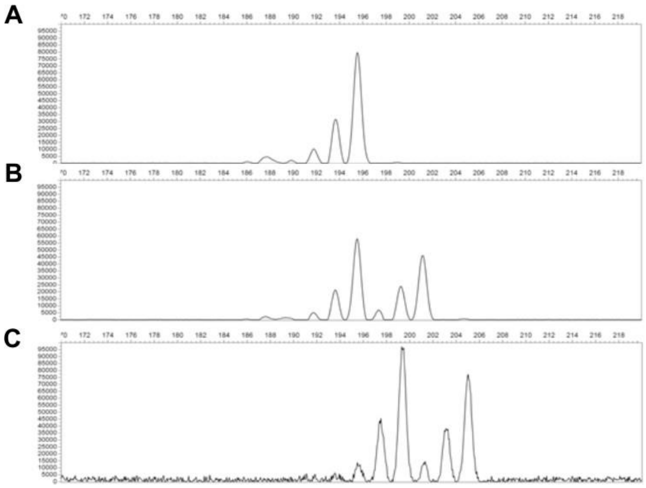

Supplemental Figure S1. (**A**) Graphic visualization of the DNA microsatellite genotyping of a proband, which due to the deletion presents only one allele, 196bp in size. (**B** and **C**) Alleles of the mother and father respectively, both heterozygous, one of the mother's alleles (196bp) has the same fragment size as the allele present in the proband (196bp), indicating that the transmitted allele to the proband comes from the mother, revealing a paternal origin of the deletion.

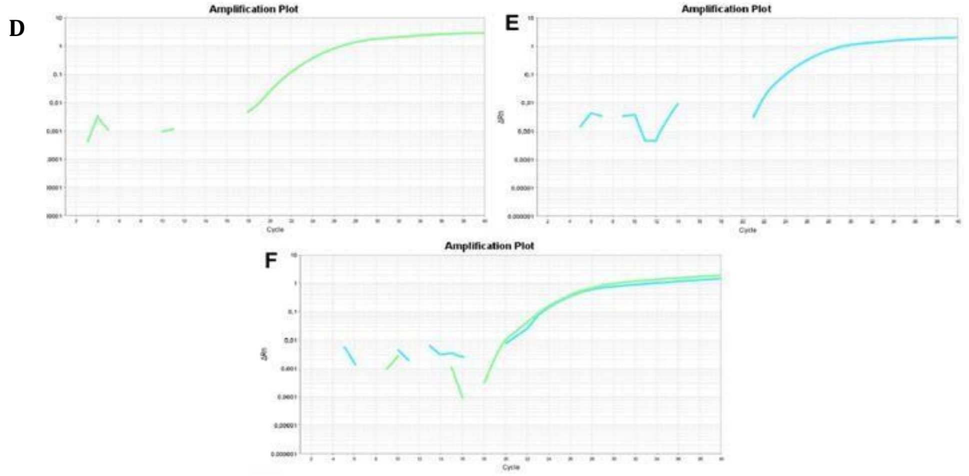

Supplemental Figure S2: (**D**) Proband's graph of allele curves obtained with qPCR, showing only one green curve, due to the deletion, determining that the proband has a C allele. (**E**) Father's graph that displays only one blue curve, indicating T/T homozygosity. (**F**) Mother's graph shows both blue and green curves, indicating C/T heterozygosity. Since only the proband and the mother exhibit the green curve, it can be inferred that the allele transmitted to the proband is of maternal origin, revealing a paternal origin of the deletion.

Supplemental Table S1: Parental origin definition by genotyping of DNA microsatellite markers and SNPs.

Family	**D22S1638**	**D22S941**	**D22S944**	**D22S1623**	**D22S264**	**rs4819519**	**rs5993650**	**Origin**
P1	2	**8**	**4**	6	**3**	C	C	
M1	7/7	13/13	5/5	6/6	2/9	C/T	C/T	MAT
F1	-	**8/16**	**4/7**	6/6	**3/9**	C/T	C/T	
P2	**4**	**13**	5	6	**3**	-	-	
M2	5/9	8/8	5/9	6/6	7/9	-	-	MAT
F2	**4/9**	**8/13**	5/5	6/6	**3/3**	-	-	
P3	**8**	8	**6**	5	**7**	**C**	C	
M3	5/7	8/8	4/5	5/6	8/9	T/T	C/C	MAT
F3	**7/8**	8/13	**5/6**	5/5	**7/10**	**C/T**	C/T	
P4	5	**13**	**4**	**6**	**7**	C	T	
M4	-	4/8	6/7	2/5	6/9	C/T	C/T	MAT
F4	-	**13/13**	**4/5**	**5/6**	**7/10**	C/T	T/T	
P5	7	**3**	**6**	-	7	C	**T**	
M5	6/7	8/16	5/7	5/6	6/7	C/T	C/C	MAT
F5	4/7	**3/8**	**5/6**	5/6	6/7	C/T	**C/T**	
P6	**8**	**3**	**4**	5	**10**	C	**T**	
M6	**5/8**	**3/13**	**4/5**	5/6	**7/10**	C/T	**T/T**	PAT
F6	2/5	8/14	5/6	1/5	3/6	C/T	C	
P7	**5**	**13**	**5**	**5**	**7**	**C**	**T**	PAT
M7	**2/5**	**13/15**	**4/5**	**5/6**	**7/8**	**C/C**	**C/T**	

P8	**6**	4	**3**	6	**8**	-	-	
M8	7	4/14	4/5	1/6	6/7	-	-	MAT
F8	**4/6**	8/12	**3/5**	5/6	**7/8**	-	-	
P9	7	8	**8**	**7**	8	C	C	
M9	7/9	8/8	4/7	5/5	8/9	C/T	C/T	MAT
F9	5/7	8/14	**4/8**	**5/7**	6/8	C/C	C/C	
P10	**7**	8	**2**	6	9	C	C	
M10	5/8	8/12	5/7	6/8	9/9	C/C	C/C	MAT
F10	**5/7**	8/9	**2/7**	5/6	2/9	C/T	C/C	
P11	**4**	12	**6**	5	**7**	-	T	
M11	3/7	12/16	4/4	5/5	2/9	C/C	C/T	MAT
F11	**3/4**	8/12	**4/6**	5/5	**7/7**	T/T	C/T	
P12	3	**3**	5	5/5	9	T	**T**	
M12	3/2	8/14	5/6	5/5	7/9	T/T	C/C	MAT
F12	3/2	**3/8**	4/5	5/6	9/11	C/T	**C/T**	
P13	**2**	**7**	5	**6**	**8**	T	C	
M13	**2/13**	**7/12**	5/5	**6/6**	**7/8**	C/T	C/T	PAT
F13	3/5	4/13	5/6	3/4	6/9	C/T	C/C	
P14	7	**11**	4	**6**	**8**	C	T	
M14	7/7	12/15	4/5	4/4	2/7	C/C	C/C	MAT
F14	6/7	**7/11**	4/5	**6/6**	**8/10**	C/C	-	
P15	**6**	**7**	**4**	**4**	**8**	C	**C**	
M15	2/7	4/12	5/5	6/6	6/10	C/C	T/T	MAT
F15	**5/6**	**7/7**	**4/4**	**4/6**	**7/8**	C/C	**C/C**	
P16	**6**	12	5	6	**2**	C	**C**	
M16	**6/6**	7/7	5/5	6/9	**2/8**	C/T	**C/C**	PAT
F16	5/7	2/12	5/10	3/6	8/8	C/C	T/T	
P17	4	7	**5**	6	**2**	**C**	**T**	
M17	2/4	7/12	**5/11**	4/6	**2/11**	**C/C**	**C/T**	PAT
F17	4/6	7/11	6/7	5/6	8/9	T/T	C/C	
P18	**2**	12	**4**	**1**	**10**	T	**T**	
M18	**2/7**	12/13	**4/5**	**1/6**	**2/10**	T/T	**T/T**	PAT
F18	5/7	5/12	5/5	4/6	7/7	T/T	C/C	
P19	7	**12**	**5**	**4**	**7**	-	-	
M19	7/7	**12/13**	**5/5**	**4/4**	**7/10**	-	-	PAT
F19	7/8	8/15	3/6	6/8	9/12	-	-	
P20	**2**	7	**6**	**4**	7	C	C	
M20	**2/2**	7/12	**6/10**	**4/6**	2/7	C/T	C/T	PAT
F20	6/6	7/12	5/5	6/6	7/9	C/C	C/T	
P21	9	**13**	**5**	5	**1**	**T**	T	
M21	9/9	**7/13**	**5/5**	5/5	**1/1**	**C/T**	C/C	PAT
F21	3/9	7/12	4/4	5/5	8/8	-	-	
P22	9	12	**5**	**4**	**1**	-	-	PAT

M22	9/9	12/12	**3/5**	**4/4**	**1/7**	-	-	
F22	9/9	12/15	3/9	5/5	8/8	C/T	C/T	
P23	9	**14**	**8**	**5**	**9**	**T**	C	
M23	9/9	**2/14**	**3/8**	**4/5**	**5/9**	**C/T**	C/T	PAT
F23	6/9	7/12	6/10	4/4	1/10	C/C	C/T	
P24	**5**	**13**	**5**	5	**11**	C	**T**	
M24	3/9	7/9	3/6	4/5	1/6	C/T	C/C	MAT
F24	**5/5**	**13/13**	**4/5**	5/5	**9/11**	C/T	**T/T**	
P25	**5**	**6**	**5**	3	11	-	C	MAT
M25	**9**	**5/8**	**3/3**	2/4	5/10	C/T	C/C	
P26	**3**	**15**	**3**	**5**	**9**	C	C	
M26	9/9	2/4	4/4	4/4	4/6	C/C	C/T	MAT
F26	**1/3**	**15/15**	**3/4**	**5/5**	**8/9**	-	-	
P27	9	**12**	**4**	4	**8**	-	-	
M27	7/9	**8/12**	**4/4**	4/4	**1/8**	C/T	C/T	PAT
F27	6/9	2/8	5/5	4/4	1/5	C/T	C/T	
P28	**5**	**13**	**5**	**5**	**10**	C	**T**	
M28	**4/5**	**13/13**	**5/5**	**5/6**	**9/10**	C/T	**T/T**	PAT
F28	1/1	7/7	4/4	4	7/8	C/C	C/C	
P29	4	**4**	5	**5**	**6**	C	T	
M29	4/4	13/13	5/5	4/6	2/7	C/T	T/T	MAT
F29	4/9	**4/13**	5/5	**5/5**	**6/9**	C/T	C/T	
P30	**7**	**8**	4	**6**	**10**	C	C	
M30	**7/7**	**8/8**	4/6	**5/6**	**9/10**	C/C	C/C	PAT
F30	9/9	9/9	4/4	5/5	7	C/C	C/C	
P31	7	**8**	4	6	**10**	C	**C**	
M31	7/7	**8/13**	4/5	6/6	**10/10**	C/C	**C/T**	PAT
F31	2/7	13/13	4/5	5/6	2/7	C/T	T/T	
P32	**4**	13	**11**	4	**6**	-	**-**	
M32	5/8	13/13	2/5	3/4	7/7	C/C	C/T	MAT
F32	**4/9**	9/13	**4/11**	4/4	**6/9**	-	C/T	
P33	**8**	**14**	**4**	**6**	**12**	C	T	
M33	**8/11**	**5/14**	**4/4**	**1/6**	**2/12**	C/C	C/T	PAT
F33	2/2	13/13	5/11	5/8	7/7	C/T	T/T	
P34	**3**	**14**	**4**	**4**	**7**	T	C	
M34	**3/3**	**13/14**	**4/5**	**4/4**	**7/7**	C/T	C/T	PAT
F34	2/7	8/13	5/5	5/5	2/2	C/T	C/T	
P35	**5**	**14**	5	6	**7**	C	T	
M35	3/6	10/13	2/5	5/6	6/9	C/T	T/T	MAT
F35	**2/5**	**14/14**	5/10	6/6	**7/7**	C/T	T/T	
P36	**7**	13	**5**	5	**7**	-	-	
M36	**7/7**	9/13	**5/5**	5/5	**7/7**	-	-	PAT
F36	5/5	13/13	7/7	5/6	6/6	-	-	

P37	8	**7**	**5**	5	5	**T**	C	PAT
M37	8/8	**7/16**	**5/5**	5/6	5/10	**C/T**	U	
F37	8/8	8/13	6/8	5/5	5/7	C/C	C/T	
P38	**4**	**13**	-	6	**7**	T	T	MAT
M38	2/7	12/14	6/11	5/6	10/10	C/T	T/T	
F38	**4/4**	**13/13**	4/4	5/6	**2/7**	T/T	T/T	
P39	**4**	**14**	**3**	**6**	7	C	T	PAT
M39	**4/7**	**14/14**	**3/4**	**6/6**	5/7	C/C	T/T	
F39	6/6	6/12	6/11	5/5	7/10	C/T	C/T	
P40	**2**	**13**	4	**5**	**6**	-	-	PAT
M40	**2/2**	**13/13**	4/5	**5/5**	**6/9**	-	-	
F40	-	-	4/4	-	10/11	-	-	
P41	7	**4**	**1**	**6**	8	T	**T**	PAT
M41	4/7	**4/4**	**1/5**	**6/6**	8/11	C/T	**T/T**	
F41	7/10	14/18	6/6	4/4	-	C/T	C/C	
P42	5	**14**	5	**3**	**6**	T	T	MAT
M42	5/7	9/13	5/11	5/5	3/7	T/T	C/T	
F42	2/5	**9/14**	5/5	**3/5**	**6/6**	T/T	C/T	
P43	7	12	**7**	**6**	10	T	T	MAT
M43	4/7	12/12	6/9	1/5	7/10	T/T	T/T	
F43	5/7	11/12	**5/7**	**5/6**	2/10	C/T	T/T	
P44	7	**14**	6	5	-	C	**T**	MAT
M44	4/7	9/17	6/8	5/5	7/10	C/T	C/C	
F44	4/7	**9/14**	6/6	5/6	7/9	C/C	**C/T**	
P45	**2**	**14**	5	**5**	**7**	C	T	PAT
M45	**2/6**	**12/14**	5/5	**1/5**	**7/7**	C/T	-	
F45	4/7	17/17	5/5	6/6	2/10	C/C	C/T	
P46	**7**	**10**	5	**5**	**9**	C	**C**	PAT
M46	**7/7**	**10/13**	4/5	**5/8**	**2/9**	C/T	**C/T**	
F46	4/5	14/14	4/5	6/8	2/10	C/T	T/T	
P47	2	14	**1**	**6**	2	T	T	PAT
M47	2/7	9/14	**1/5**	**6/6**	2/9	C/T	C/T	
F47	2/4	14/14	6/6	4/4	2/2	-	C/T	
P48	**8**	**9**	**4**	6	**11**	T	C	PAT
M48	**5/8**	**6/9**	**4/6**	3/6	**6/11**	T/T	C/C	
F48	7	14/14	2/7	5/6	2/9	C/T	C/T	
P49	**6**	**9**	**6**	**5**	-	**C**	C	MAT
M49	7/7	4/4	5/5	4/4	8/10	T/T	-	
F49	**6/7**	**9/15**	**5/6**	**5/5**	6/7	**C/T**	C/C	
P50	**9**	**6**	**7**	6	**6**	C	C	MAT
M50	**7/7**	**15/17**	**4/5**	6/6	**9/10**	C/T	C/T	
P51	**6**	14	**9**	**6**	-	T	T	MAT
M51	**4/5**	14/17	**5/5**	**5/5**	6/6	T/T	C/T	

P52	7	**15**	4	6	**7**	**T**	T	MAT
M52	7/9	**1/1**	4/5	5/6	**2/9**	**C/C**	C/T	
P53	**7**	**14**	**5**	**5**	**6**	**C**	**T**	PAT
M53	**2/7**	**4/14**	**5/5**	**5/5**	**6/10**	**C/T**	**T/T**	
P54	**7**	**10**	**7**	**6**	9	**C**	**C**	PAT
M54	**7/8**	**10/15**	**6/7**	**6/8**	8	**C/C**	**C/T**	
P55	**7**	15	**5**	**4**	**9**	**C**	**C**	PAT
M55	**7/7**	14/16	**5/5**	**4/4**	**2/9**	**C/T**	**C/T**	
P56	**7**	**9**	**6**	**5**	**8**	-	**T**	PAT
M56	**2/7**	**9/14**	**4/6**	**5/5**	**6/8**	C/T	**T/T**	
P57	**4**	**9**	5	**5**	**6**	C	C	MAT
M57	**5/7**	**12/14**	4/5	**4/6**	**8/10**	C/T	C/C	
P58	**6**	**16**	**6**	**4**	**10**	**T**	C	PAT
M58	**6/8**	**15/16**	**2/6**	**4/5**	**9/10**	**T/T**	-	
P59	7	9	**11**	**9**	10	C	C	MAT
M59	5/7	9/14	**6/7**	**6/6**	6/10	C/T	C/T	
P60	**5**	**14**	**4**	6	**8**	-	-	MAT
M60	**7/12**	**9/9**	**5/6**	3/6	**7/10**	T/T	C/C	
P61	**2**	**4**	**5**	**5**	**7**	**C**	-	PAT
M61	**2/6**	**4/9**	**5/5**	**5/6**	**2/7**	**C/C**	-	

(P) proband, (M) mother, and (F) father; (MAT) Maternal, (PAT) paternal. D22S1638, D22S941, D22S944, D22S1623, D22S264 are microsatellite DNA markers and rs4819519 and rs5993650 are SNP assays. Each allele of the microsatellite markers was numbered according to the fragment size.
